# Microsatellite instability in colorectal cancer is associated with local lymphocyte infiltration and low frequency of distant metastases

**DOI:** 10.1038/sj.bjc.6602534

**Published:** 2005-04-26

**Authors:** A Buckowitz, H-P Knaebel, A Benner, H Bläker, J Gebert, P Kienle, M von Knebel Doeberitz, M Kloor

**Affiliations:** 1 Department of Surgery, University Hospital of Heidelberg, Heidelberg, Germany; 2 Central Unit Biostatistics, German Cancer Research Center (DKFZ), Heidelberg, Germany; 3 Institute of Pathology, University of Heidelberg, Heidelberg, Germany; 4 Institute of Molecular Pathology, University of Heidelberg, Im Neuenheimer Feld 220/221, 69120 Heidelberg, Germany

**Keywords:** colorectal cancer, Crohn's like reaction, microsatellite instability, lymphocyte infiltration, organ metastasis, hereditary non-polyposis colorectal cancer (HNPCC)

## Abstract

Colorectal carcinomas (CRCs) with high microsatellite instability (MSI-H) share clinicopathological features distinctly different from their microsatellite stable (MSS) counterparts. Unlike MSS cancers, MSI-H CRCs occur predominantly in the right-sided colon and are often characterised by a strong lymphocyte infiltration. A poor differentiation pattern is found in most MSI-H CRCs, even though patients with MSI-H carcinomas seem to have a significantly longer survival after surgical resection. To clarify which factors contribute to the obvious paradoxon of a more favourable prognosis of MSI tumours, several clinical and histopathological features as well as the microsatellite status were evaluated in 120 colorectal cancer cases fulfilling clinical criteria (Bethesda) indicative for familial colorectal cancer. Microsatellite instablity status and lymphocyte infiltration were related to tumour stage and patients' follow-up. Statistical analysis confirmed well-known relations, such as enhanced lymphocyte infiltration accompanied by Crohn's like reaction (CLR) in MSI-H cancers (CLR+ in 27 out of 47 MSI-H *vs* 14 out of 71 MSS CRCs, *P*<0.001). However, after stratification for depth of local invasion and penetration of the primary tumour, T3 tumours displaying MSI had a significantly lower rate of distant metastases (M1 in four out of 35 MSI-H *vs* 20 out of 41 MSS CRCs, *P*<0.001). A similar tendency was observed for CLR-positive CRCs (M1 in six out of 29 CLR+ *vs* 17 out of 45 CLR− CRCs, *P*=0.13). In a logistic regression model, the MSI-H phenotype and the presence of CLR were independent predictors of a low UICC stage (*P*=0.006 and 0.04, respectively). These data, together with the recent definition of highly immunogenic neo-antigens expressed in MSI-H tumour cells, suggest that MSI-H CRCs elicit a protective host response that may prevent metastasis formation.

Colorectal cancer is one of the most common causes of cancer-related deaths in the Western world. Even though preventive cancer screening contributes to a decrease of colorectal cancer incidence in the elderly, recent reports indicate increasing rates in the younger population (O'Connell *et al*, 2003). Colorectal cancer remains a major health problem causing significant morbidity and mortality, especially in industrialised countries. In Germany, about 57 000 novel cases of colorectal cancer are recorded each year (Becker, 2003).

Colorectal carcinomas (CRCs) generally arise from precancerous lesions by accumulation of mutations in genes coding for proteins involved in controlling cell growth and differentiation. For the development of colorectal cancer, two major pathways have been described. The more common pathway is characterised by large genomic rearrangements (Lengauer *et al*, 1997), which are found in about 85% of colon cancers. An exemplary multistep model, which is called the chromosomal instability (CIN) pathway, has been described by Fearon and Vogelstein (1990). According to this schematic process, the inactivation of the APC tumour suppressor gene is a key event in early tumorigenesis, whereas mutations of the TP53 gene occur at a late stage and thus contribute to the development of a malignant phenotype and invasive tumour growth. A second pathway is characteristic for patients with the autosomal-dominant hereditary non-polyposis colorectal cancer (HNPCC) syndrome, a condition which is found in 2–5% of all colorectal cancer patients. Hereditary non-polyposis colorectal cancer familial cancer syndrome is caused by germline mutations in genes coding for proteins of the DNA mismatch repair (MMR) system, hMLH1 and hMSH2 are most frequently affected (Boland (2000) for a review see Lynch and de la Chapelle, 2003). Defects in the MMR system lead to the accumulation of mutations at short repetitive DNA sequences, termed microsatellites. Most of them are localised in noncoding regions of the genome. However, certain growth-inhibiting or proapoptotic genes, for example, *TGFbRII, BAX, hMSH3* and *hMSH6*, harbour microsatellites within their coding sequence (Markowitz *et al*, 1995; Malkhosyan *et al*, 1996; Woerner *et al*, 2003). Insertion/deletion mutations at these loci cause translational frameshifts and lead to a loss of function of the corresponding proteins, thereby contributing to the progression of neoplastic transformation (Furlan *et al*, 2002). The microsatellite instability (MSI) pathway also contributes to about 15% of sporadic CRCs. In most MSI cases without family history of cancer, promoter methylation of the *hMLH1* gene is supposed to be the underlying mechanism (Kane *et al*, 1997; Miyakura *et al*, 2001). Although recent work indicates that there are overlaps of the CIN and MSI pathways in a considerable number of cases (Goel *et al*, 2003), the consideration of MSI and CIN as separate mechanisms of tumorigenesis seems well justified.

Colorectal carcinoma of the MSI phenotype displays several clinicopathological features distinctly different from those found in CIN CRC. High-grade microsatellite unstable (MSI-H) colon tumours predominantly have a right-sided location and frequently present with a strong lymphocyte infiltration (Smyrk *et al*, 2001). The presence of characteristic lymphocyte aggregations, the so-called Crohn's like reaction (CLR, Graham and Appelman, 1990), has also been reported to be more frequent in MSI-H colon cancer (Alexander *et al*, 2001; Greenson *et al*, 2003). Many MSI-H carcinomas display a poor differentiation pattern, often with mucinous or signet cell areas (Greenson *et al*, 2003). Data on the prognosis of MSI-H CRC are conflicting. Salahshor *et al* (1999) found an obvious trend towards a better outcome of their MSI-H collective, but this was not statistically significant. Feeley *et al* (1999) could not see any impact of microsatellite status on patients' survival at all, but the validity of this study is limited by the low number of MSI-H cases. In summary, however, most studies revealed a longer disease-free and overall survival for patients with MSI-H CRC compared to MSS CRC (Watson *et al*, 1998; Gryfe *et al*, 2000; Samowitz *et al*, 2001).

For a better prognostic assessment, Dolcetti *et al* (2002) suggest the combination of both the evaluation of local lymphocytosis and the microsatellite status; MSI in combination with a high content of intraepithelial cytotoxic lymphocytes was found to be related with an improved overall survival in a group of exclusively right-sided CRCs (Guidoboni *et al*, 2001).

To determine the relative impact of microsatellite status and antitumoural lymphocyte reaction on the course of disease, we analysed histological and molecular features of CRCs in patients clinically suspicious of HNPCC and compared them with tumour stage and overall survival of the patients.

## MATERIALS AND METHODS

### Patients' characteristics

Tumour specimens from 120 patients treated for colorectal cancer in the Department of Surgery, University Hospital of Heidelberg, from 1992 to 2002 were included in this prospective study. The study collective consists of 120 samples consecutively obtained for microsatellite analysisin the Institute of Molecular Pathology, University of Heidelberg. Histopathological assessment, microsatellite analysis and genetic testing were performed with informed consent of all patients. Only patients fulfilling Amsterdam or Bethesda criteria (Table 1, Vasen *et al*, 1991; Rodriguez-Bigas *et al*, 1997; Boland *et al*, 1998), that is, patients suspicious of HNPCC, were included in this study. From 120 patients included, 21 (17.5%) fulfilled Amsterdam criteria, among the remaining 99 (82.5%), 23 (19.2%) fulfilled Bethesda 2, three (2.5%) Bethesda 3 and 73 (60.8%) Bethesda 4 criteria.Of these, 66 (55.0%) of the patients were male, 54 (45.0%) were female. In all, 20 patients had from two (14 patients) to six (one patient) synchronous CRCs. Patients diagnosed with synchronous tumours in extracolonic localisation were generally excluded. 5-Fluorouracil (5-FU) chemotherapy was administered to nine patients with UICC stage II, 18 patients with UICC stage III and 24 patients with UICC stage IV. Chemotherapeutic regimens were recorded for all patients included.

### Tissue preparation

Surgically resected colonic cancer samples were fixed in formalin (4–10%) and embedded in paraffin. Tissue sections were prepared for DNA isolation and subsequent microsatellite marker amplification (10 *μ*m) as well as for pathohistological analysis (2 *μ*m).

### Microsatellite analysis

DNA was isolated from microdissected tumour and normal tissue using the Qiagen DNeasy Tissue Kit (Qiagen, Hilden, Germany). Microsatellite status was determined using 5–10 markers from the standard ICG-HNPCC marker panel (Boland *et al*, 1998). PCR products were amplified with fluorescein-labelled oligonucleotide primers as described previously (Sutter *et al*, 1999). Labelled PCR products were separated on an ABI3100 analyser (Applied Biosystems, Darmstadt, Germany), results were analysed using the GeneScan software version 3.7 (Applied Biosystems) as previously described (Woerner *et al*, 2003).

According to the recommendations of the National Cancer Institute (1998), tumours with instability at 30% or more of the tested microsatellite loci were classified as highly microsatellite unstable (MSI-H), those with instability at less than 30% of tested markers as low microsatellite unstable (MSI-L), and tumours with stability in all tested loci as microsatellite stable (MSS) (Boland *et al*, 1998). Low microsatellite unstable and MSS tumours were combined as non-MSI-H according to previous studies (Ribic *et al*, 2003) and are referred to as ‘MSS’ in this paper.

### Pathohistological evaluation

**Figure 1:**
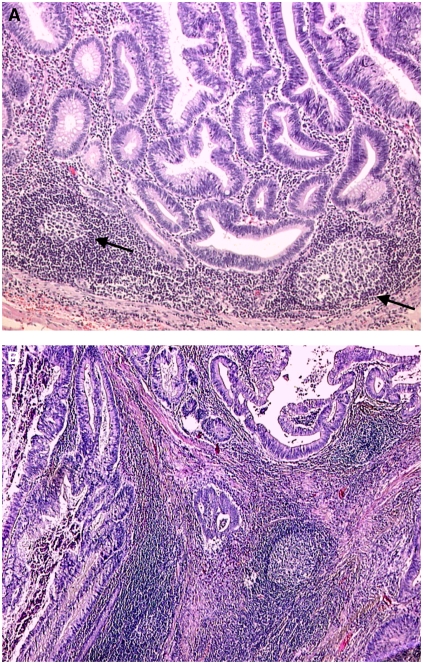
Histological evaluation of Crohn's like reaction. HE-stained sections of MSI-H tumours with marked lymphocyte infiltration. (**A**) Exemplary illustration of Crohn's like lesions (black arrows) with apparent germinal centres surrounding the invasion front of the tumour (× 100 magnification). (**B**) Peritumoural lymphocytes surrounding the penetration border of the primary tumour like a cap (× 50). Note the pronounced Crohn's like lymphoid reaction. Germinal centres that were not mandatory for the diagnosis of CLR are clearly detectable in both tumour sections.

In all, 2 *μ*m tissue sections were deparaffinised and stained with hematoxylin and eosin (HE) following standard protocols. Antitumoural lymphoid reaction and presence of a CLR were evaluated in HE-stained sections from 1 to 3 paraffin blocks of the same tumour (Figure 1). The recommendations of the German HNPCC Consortium were applied (Ruschoff *et al*, 2004). The presence or absence of CLR was scored as 1 or 0, repsectively. For a positive score, at least three nodular lymphocyte aggregations had to be detectable in the sample (Figure 1), the presence of germinal centres was not mandatory. In two cases, CLR presence could not be evaluated, because the tumour invasion front was not detectable in the paraffin-embedded material.

Histological evaluation was carried out by three observers (AB, HB, MK) not aware of MSI status or tumour stage. In patients with synchronous tumours, the most advanced tumour was used for the analysis. In eight of 20 cases with synchronous carcinomas, 2–6 different tumours were evaluated. Without exception, scoring was the same for all synchronous tumours (data not shown).

### Tumour staging

For staging, the UICC/AJCC TNM system was applied (AJCC, 1997).

### Statistical analysis

The primary end point for the analysis was survival time from the date of first tumour diagnosis. The median follow-up for survival was calculated according to the method of Korn (1986). Pairwise comparisons of the distributions of categorical data were performed using Fisher's exact test. The 95% confidence intervals (CI95) for binomial probabilities were computed using the score-test-based approach of Wilson (Agresti and Coull, 1998). The pairwise comparison of the distribution of age at diagnosis was performed using the Wilcoxon rank sum test and for the pairwise comparison of survival time distributions the log-rank test was applied. Estimation of survival time distributions was performed by the method of Kaplan and Meier. A Cox proportional hazards regression was used to identify independent prognostic factors for survival. The model included microsatellite status together with UICC stage, CLR, and age at diagnosis and 5-FU chemotherapy as possible prognostic factors. Ordinal logistic regression was used to model the effect of microsatellite status together with CLR and age at diagnosis on UICC stage. UICC stages I and II were combined for the regression analyses. Assuming ‘missing at random’, missing value imputation was performed for two missing values of CLR. An effect was judged as statistically significant at a *P*-value not larger than 5%. To provide quantitative information on the relevance of the results 95CI of odds ratios (OR) and hazard ratios (HR) were computed. The statistical analyses were performed by the statistical software package R, version 1.8.1 (R Development Core Team, 2003) together with the Design software library (Harrell, 2001).

## RESULTS

### Microsatellite status and associated characteristics

Microsatellite analysis of the tumour samples from 120 Bethesda-positive patients examined in this study revealed an MSI-H phenotype in 47 (39.2%) cases, 73 (60.8%) were non-MSI-H (64 MSS, nine MSI-L). Patients in the MSI-H group (median 39 years) were ‘younger’ than patients in the MSS collective (median 43 years, exact Wilcoxon's rank sum test: *P*=0.02); the median age at time of first diagnosis was 42 years (Table 2).

Clinical criteria were distributed unequally among MSI-H and MSS group (Fisher's exact test: *P*=0.01). The most obvious discrepancy was observed for Amsterdam-positive cases, which were much more frequent in the MSI-H (14/47, 30.1%) than in the MSS group (7/73, 9.6%, Fisher's exact test: *P*=0.006, Table 2).

The trend towards a more advanced tumour stage observed in the MSS CRC group (Fisher's exact test: *P*<0.001, Table 2) is reflected by the higher frequency of patients treated with 5-FU chemotherapy (Fisher's exact test: *P*=0.04, Table 2).

For comparison of tumour localisation with microsatellite status, only patients with single carcinomas (*n*=100) were evaluated. As expected, right-sided location correlated closely with the MSI-H phenotype. Among 30 carcinomas in the proximal colon, 20 were MSI-H (66.7%), in the group of tumors with distal location, only 18 of 70 tumours (25.7%) displayed the MSI-H phenotype (Fisher's exact test: *P*<0.001, Table 2).

In all, 27 of 47 MSI-H carcinomas had a CLR (57.4%), whereas CLRs were found in only 14 from 71 MSS tumours (19.7%). Thus, CLR frequency was significantly elevated in MSI-H colorectal cancers (Fisher's exact test: *P*<0.001, Table 2).

### Patients' overall survival

**Figure 2:**
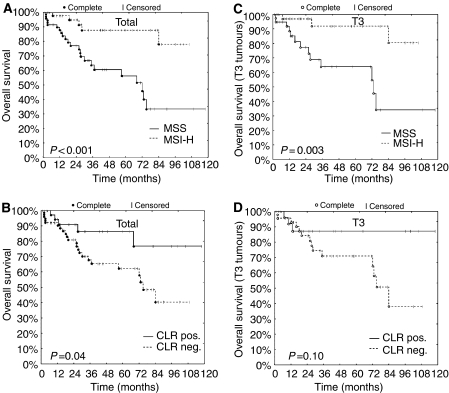
Kaplan–Meier estimates of overall survival. (**A** and **B**) Patients of all tumour stages stratified by microsatellite status (**A**) and presence of Crohn's like reaction (CLR, **B**). (**C** and **D**) Overall survival of patients with T3 stage colon cancer depending on microsatellite status (**C**) and CLR (**D**). *P*-values shown were calculated using the log-rank test for survival differences.

The median follow-up was 33 months from the date of first diagnosis, 29 patients died within the follow-up period. The survival distribution of MSI-H patients differed significantly from that of MSS patients (log-rank test: *P*<0.001, Table 2). Kaplan–Meier estimates of the survival time distributions are presented in Figure 2. The estimated 5-year survival rate of MSI-H patients was 88% (CI95: 77–100%) compared to 56% (CI95: 42–75%) in the MSS group (Figure 2A; log-rank test: *P*<0.001). An improved overall survival was also observed in patients with CLR+CRC (Figure 2B, *P*=0.04). No significant differences were observed in groups with or without 5-FU chemotherapy (not shown). Additionally, we tested the prognostic value of microsatellite status together with UICC stage, presence of CLR, and age at diagnosis and application of 5-FU chemotherapy by using a proportional hazards regression model. Here, UICC stage and age at diagnosis were the only statistically significant prognostic factors for overall survival (Table 3).

The MSI-H phenotype was rarely detected in stage UICC IV: only six out of 43 (13.9%) tumours with distant metastases (UICC IV) were MSI-H, compared to 41 out of 77 (53.2%) in the collective with UICC I-III (Fisher's exact test: *P*<0.001, Table 2). For presence of CLR, the observed tendency was similar. From 42 UICC IV cases that could be scored for lymphocyte infiltration at primary tumour site, only seven (16.7%) primary tumours had a marked peritumoural lymphocyte infiltration with CLR, whereas in the collective with UICC grade I–III the relation of CLR-positive tumours was 35 out of 76 (46.1%, Fisher's exact test: *P*=0.001).

Comparison of overall survival after stratification by microsatellite status and presence of CLR is shown in Figure 2C and D for tumours infiltrating the lamina muscularis propria (all other T stages did not reveal significant results). A lower risk was observed for patients with MSI-H T3 carcinomas (log-rank test: *P*=0.003, Figure 2C) but this result could not be confirmed by multiple proportional hazards regression analysis (Table 3).

### Metastasis frequency in correlation to microsatellite status and CLR

These results raised the question whether the longer overall survival of patients with MSI-H tumours was linked to the occurrence of organ metastases. To address this question in more detail, we stratified the tumours by depth of infiltration and compared the occurrence of metastases in the MSI-H *vs* the MSS group. From 76 T3 carcinomas, 35 (46.1%, CI95: 0.35–0.57) were MSI-H, hence the MSI-H frequency was comparable to the MSI-H frequency observed overall in all included patients (39.2%, CI95: 0.31–0.48).

T3 cancers with MSI-H had organ metastases defining UICC IV stage in 4/35 cases (11.4%), while those with MSS phenotype in almost half of the cases (20 out of 41, 48.8%, Table 4). The increased frequency of metastases in MSS T3 cases was highly significant (Fisher's exact test: *P*<0.001, Table 4).

To clarify whether this observation was related to morphological features of an antitumoural immune response, T3 tumours were grouped according to the presence of a CLR. The comparison of M stage in T3 carcinomas with or without CLR revealed a trend towards a lower frequency of metastases in cases with CLR (six out of 29 M1 in CLR+ T3 tumours *vs* 17 out of 45 M1 in CLR−, *P*=0.13, Table 4).

In summary, we observed a markedly reduced frequency of organ metastases in T3 colorectal cancers that exhibited either the MSI-H phenotype, a CLR or both.

In regard to lymph node metastases, differences were neither significant after stratification for microsatellite status nor for CLR presence (*P*=0.28 and 0.51, respectively, Table 4).

To test microsatellite status and CLR as predictors of UICC tumour stage, a logistic regression model was applied (Table 5). For CLR, the statistically significant correlation was limited to the regression analysis that included tumours of all T stages (OR 0.44, *P*=0.04). In contrast, MSI-H was significantly associated with a lower UICC stage when looking at all tumours (OR 0.33, *P*=0.006) and also in the T3 subgroup (OR 0.24, *P*=0.006). These data show that the improved overall survival of patients with MSI-H CRC included in this study is closely linked to lower rates of distant metastases, even after stratification for local depth of invasion of the primary tumour.

## DISCUSSION

In this study, we analysed the impact of MSI and Crohn's like lymphoid reaction on the course of disease in 120 colorectal cancer patients fulfilling clinical criteria suggesting HNPCC. Our inclusion criteria resulted in a cohort of patients with an age at diagnosis younger than in a random population of patients with colon cancer. This strategy significantly increases the number of MSI-H cases. Focusing on Bethesda-positive patients results in a stratification that may provide a better comparability of MSI-H and MSS cases, as clinical differences between the two collectives are reduced. It is important to note, that none of the included patients were from families in which HNPCC had previously been confirmed. Therefore, a statistical bias towards an earlier detection of MSI cases due to an intensified screening of persons at risk can be excluded. Furthermore, MSI status had no influence on operative or perioperative treatment as this was generally not known before the time of operation. All patients received a standardised oncologic colorectal resection in the No-Touch-Isolation technique originally described by Turnbull (1975) unless when in a palliative noncurative situation.

When comparing MSS and MSI-H cases, significant differences were found for distribution of clinical criteria, tumour localisation, UICC stage of disease, M stage, vital status of patients and presence of CLR.

Not surprisingly, the predictive value for microsatellite unstable tumours was highest for Amsterdam criteria. In two-thirds of Amsterdam positive cases tumours were diagnosed as MSI-H. For our further analyses, no discrimination was made between sporadic cases and cases with a positive family history as the microsatellite status of the tumour itself was believed to be crucial for its histological appearance and biological behaviour. This approach is supported by a large study comparing HNPCC-associated and sporadic MSI-H CRCs where no significant histopathological differences were found, except a marginally higher frequency of mucinous tumours in cases with positive family history (Shia *et al*, 2003).

To analyse the antitumoural immune reaction of the host, we focused on the presence of a CLR and not on the density of intratumoural lymphocytes. This strategy was chosen to directly address the stromal inflammatory reaction. Furthermore, the interobserver reproducibility has been reported to be much better for the assessment of CLR than for counting of tumour-infiltrating lymphocytes (Alexander *et al*, 2001). Moreover, preliminary data with a limited number of tumour samples indicated a good correlation of CLR with a high density of tumor-infiltrating cytotoxic (CD8+) lymphocytes, supporting the hypothesis that CLR positivity is indeed associated with a cytotoxic antitumoural immune response.

As in previous reports, we could show that the presence of a CLR was closely associated with a microsatellite unstable phenotype of colorectal cancer (Gafa *et al*, 2000). The strong antitumoural lymphoid reaction, which frequently occurs in MSI-H colorectal cancers, is commonly attributed to tumour-specific neopeptides generated during MSI-H carcinogenesis. Functional *in vitro* assays proved the immunogenicity of certain MSI-associated frameshift peptides (Linnebacher *et al*, 2001; Saeterdal *et al*, 2001; Ripberger *et al*, 2003). Both features, MSI-H status and CLR, have been reported to be associated with longer survival times in previous studies. When analysing the overall survival in our total collective stratified for microsatellite status or Crohn's like lymphoid reaction, the prognosis of patients with tumours displaying MSI-H and/or CLR was significantly better. After having found the differences in survival and the low frequency of MSI-H in UICC IV tumours, we hypothesised that the above prognostic advantage of MSI-H tumours was, at least in part, due to a reduced metastatic potential of these. Therefore, we stratified the CRCs for penetration depth of primary tumour and compared the frequency of distant metastases, which proved to be significantly lower in the MSI-H group. Similar observations had previously been reported for CRC patients with family history of HNPCC (Watson *et al*, 1998), however tumours were not tested for MSI, and sporadic CRC cases were chosen as a control group.

The observation that microsatellite status was an independent predictor of distant metastases suggests either a reduced tendency of MSI-H tumour cells to form metastases, a better antimetastatic response of the host in MSI-H cases, or both. To further examine the role of inflammatory reaction in this context, T3 cancers were grouped for CLR status and frequencies of metastases were compared. Here, a clear trend towards fewer metastases in CLR positive cases was observed. One might speculate that the lack of statistical significance merely reflects variability in CLR scoring which is more pronounced than in MSI testing. Moreover, CLR assessment only refers to morphological aspects, yet not to functional features of tumour infiltrating lymphocytes. An effective antitumoural immune reaction of the host may not necessarily be linked to density of tumour-infiltrating lymphocytes or presence of CLR. The fact, that MSI-H status is much more closely linked to fewer distant metastases than lymphoid reaction, raises the question whether there are additional features of microsatellite unstable cells that contribute to a lower metastatic potential, e.g. mutations in a number of metastasis-promoting genes. However, the presence of an antimetastatic immune protection in MSI-H CRC patients may explain recent findings that adjuvant 5-FU chemotherapy has no beneficial or even adverse effects in this collective (Ribic *et al*, 2003; Carethers *et al*, 2004). Further functional studies are needed to clarify the molecular mechanisms underlying these phenomena. This hypothesis cannot be addressed in our study, because 5-FU chemotherapy was not administered in the framework of a controlled trial. Larger prospective studies are required to clarify the clinical consequences of 5-FU therapy in MSS or MSI-H CRC patients and the mechanisms underlying these phenomena.

In summary, this study shows for the first time that MSI-H and CLR+ CRCs from Bethesda-positive patients are associated with fewer distant metastases, even in the case of deep local invasion of the primary tumour. Our data suggest a protective role of functionally active lymphocytes directed against MSI-H CRCs, which may prevent tumour cell dissemination and metastasis formation in distant organs. This observation could be explained by frameshift mutations occurring within the coding region of different genes, which lead to tumour-specific neopeptides in MSI-H tumours. This makes vaccination against these neopeptides a promising approach for novel adjuvant treatment strategies in patients with MSI-H tumours. In MMR gene mutation carriers, a preventive vaccine with a set of predictable neopeptides frequently affected during MSI tumorigenesis could inhibit or at least delay the onset of MMR-deficient tumours. In conclusion, due to its prognostic and potentially therapeutic implications MSI screening should be considered as a routine diagnostic service in all colorectal cancer cases in the future.

## Figures and Tables

**Table 1:** Clinical criteria indicative for HNPCC

**Clinical criteria for HNPCC**
*Amsterdam*
At least three family members with histologically verified colorectal cancer
One case a first degree relative
At least two successive generations affected
At least one case diagnosed before the age of 50
Exclusion of FAP

*Bethesda 2*
Synchronous or metachronous colorectal carcinomas or HNPCC-related cancers (endometrium, ovary, stomach, bile duct, small bowel, urothelium)

*Bethesda 3*
Colorectal cancer and one first degree relative with colorectal cancer or HNPCC-related cancer (diagnosis before the age of 45) and/or colorectal adenoma (diagnosis before the age of 40)

*Bethesda 4*
Colorectal or endometrial cancer, diagnosed before the age of 45

**Table 2:** Characteristics of the 120 included colorectal cancer patients

	**All patients (*n*=120)**	**Patients with MSI-H tumours (*n*=47)**	**Patients with MSS tumours (*n*=73)**	***P*-value**
Age (year, median)	42	39	43	0.02

*Clinical criteria*				
Amsterdam	21 (17.5)	14 (30.1)	7 (9.6)	0.01
Bethesda 2	23 (19.2)	10 (21.3)	13 (17.8)	
Bethesda 3	3 (2.5)	0 (0.0)	3 (4.1)	
Bethesda 4	73 (60.8)	23 (48.9)	50 (68.5)	

*Treatment*				
5-FU	51 (42.5)	14 (29.8)	37 (50.7)	
no 5-FU	69 (57.5)	33 (70.2)	36 (49.3)	0.04

*Localisation*				
Proximal colon	30 (25.0)	20 (42.6)	10 (13.7)	<0.001^a^
Distal	70 (58.3)	18 (38.3)	52 (71.2)	
Multiple	20 (16.7)	9 (19.1)	11 (15.1)	

*Stage of disease (UICC)*				
I	10 (8.3)	2 (4.3)	8 (11.0)	<0.001
II	36 (30.0)	23 (48.9)	13 (17.8)	
III	31 (25.8)	16 (34.0)	15 (20.5)	
IV	43 (35.8)	6 (12.8)	37 (50.7)	

*Primary tumour*				
T1	8 (6.7)	1 (2.1)	7 (9.6)	0.08
T2	13 (10.8)	2 (4.3)	11 (15.1)	
T3	76 (63.3)	35 (74.5)	41 (56.2)	
T4	23 (19.2)	9 (19.1)	14 (19.2)	

*Lymph node status*				
N0	56 (46.7)	26 (55.3)	29 (39.7)	0.25
N1	21 (17.5)	8 (17.0)	15 (20.5)	
N2	43 (35.8)	13 (27.7)	29 (39.7)	

*Distant metastases*				
M0	77 (64.2)	41 (87.2)	36 (49.3)	<0.001
M1	43 (35.8)	6 (12.8)	37 (50.7)	

*Crohn's like reaction*				
Absent (0)	76 (64.4)	20 (42.6)	56 (78.9)	<0.001
Present (1)	42 (35.6)	27 (57.4)	15 (21.1)	
Survival (months, median)	Infinity^b^	Infinity^c^	72	<0.001

a Comparison of proximal *vs* distal tumours, excluding multiple tumours.

b Last patient died at 84 months (est. survival probability=0.53).

c Last patient died at 84 months (est. survival probability=0.78).

Numbers in brackets indicate percentage values.

**Table 3:** Cox's proportional hazards regression for survival

**Factor**	**Effect**	**Hazard ratio (95% confidence limits)**	***P*-value**
*All data*			
Age at diagnosis (year)	10 years difference	1.67 (1.28–2.17)	<0.001
MSA	MSI-H *vs* MSS	0.45 (0.15–1.36)	0.16

UICC			0.002
	III *vs* I/II	9.22 (1.02–83.12)	
	IV *vs* I/II	28.77 (3.65–226.66)	

CLR	Present *vs* absent	0.75 (0.27–2.12)	0.59
5-FU chemotherapy	Yes *vs* no	0.59 (0.26–1.34)	0.21

*T 3 carcinomas*			
Age at diagnos is (year)	10 years difference	1.91 (1.29–2.84)	0.001
MSA	MSI-H *vs* MSS	0.44 (0.09–2.09)	0.30

UICC			0.009
	III *vs* II	6.39 (0.65–62.71)	
	IV *vs* II	23.13 (2.62–204.16)	

CLR	Present *vs* absent	0.49 (0.11–2.22)	0.35
5-FU chemotherapy	Yes *vs* no	1.55 (0.53–4.53)	0.42

**Table 4:** Frequency of metastases in patients with T3 colorectal cancers stratified by microsatellite status and presence of Crohn's like reaction

	**All T3 tumours (*n*=76)**	**T3 tumours MSI-H**	**T3 tumours MSS**	***P*-value**	**T3 tumours CLR positive**	**T3 tumours CLR negative**	***P*-value**
*Lymph n ode status*							
N0	36 (47.4)	20 (57.1)	16 (39.0)	0.28	16 (57.1)	20 (43.4)	0.51
N1	14 (18.9)	5 (14.3)	9 (22.0)		4 (14.3)	9 (19.6)	
N2	26 (35.1)	10 (28.6)	16 (39.0)		8 (28.6)	17 (37.0)	

*Distant metastases*							
M0	52 (68.4)	31 (88.6)	21 (51.2)	<0.001	23 (79.3)	28 (62.2)	0.13
M1	24 (31.6)	4 (11.4)	20 (48.8)		6 (20.7)	17 (37.8)	

**Table 5:** Ordinal logistic regression for UICC

**Factor**	**Effect**	**Odds ratio (95% confidence limits)**	***P*-value**
*All data*			
*Age at diagnosis (year)*	10 years difference	0.88 (0.67–1.16)	0.36
*MSA*	MSI-H *vs* MSS	0.33 (0.15–0.72)	0.006
*CLR*	Present *vs* absent	0.44 (0.19–0.98)	0.04

*T 3 carcinomas*			
*Age at diagnosis (year)*	10 years difference	0.70 (0.50–0.98)	0.04
*MSA*	MSI-H *vs* MSS	0.24 (0.08–0.66)	0.006
*CLR*	Present *vs* absent	0.93 (0.33–2.60)	0.88

## References

[bib1] Agresti A, Coull BA (1998) Approximate is better than ‘exact’ for interval estimation of binomial proportions. Am Stat 52: 119–126

[bib2] Alexander J, Watanabe T, Wu TT, Rashid A, Li S, Hamilton SR (2001) Histopathological identification of colon cancer with microsatellite instability. Am J Pathol 158: 527–535 11159189 10.1016/S0002-9440(10)63994-6 PMC1850324

[bib3] American Joint Committee on Cancer (1997) AJCC Cancer Staging Manual. Philadelphia: Lippincott-Raven

[bib4] Becker N (2003) [Epidemiology of colorectal cancer]. Radiologe 43: 98–104 12624666 10.1007/s00117-003-0868-9

[bib5] Boland CR (2000) Molecular genetics of hereditary nonpolyposis colorectal cancer. Ann N Y Acad Sci 910: 50–59, discussion 59–61 10911905 10.1111/j.1749-6632.2000.tb06700.x

[bib6] Boland CR, Thibodeau SN, Hamilton SR, Sidransky D, Eshleman JR, Burt RW, Meltzer SJ, Rodriguez-Bigas MA, Fodde R, Ranzani GN, Srivastava S (1998) A National Cancer Institute workshop on microsatellite instability for cancer detection and familial predisposition: development of international criteria for the determination of microsatellite instability in colon cancer. Cancer Res 58: 5248–5257 9823339

[bib7] Carethers JM, Smith EJ, Behling CA, Nguyen L, Tajima A, Doctolero RT, Cabrera BL, Goel A, Arnold CA, Miyai K, Boland CR (2004) Use of 5-fluorouracil and survival in patients with microsatellite-unstable colorectal cancer. Gastroenterology 126: 394–401 14762775 10.1053/j.gastro.2003.12.023

[bib8] Dolcetti R, Guidoboni M, Viel A, Boiocchi M (2002) Correspondence re: Samowitz *et al* Microsatellite instability in sporadic colon cancer is associated with an improved prognosis at the population level. Cancer Epidemiol Biomark Prev 10: 917–923, 2001. Cancer Epidemiol Biomarkers Prev 11: 499; author reply 499–500 12010867

[bib9] Fearon ER, Vogelstein B (1990) A genetic model for colorectal tumorigenesis. Cell 61: 759–767 2188735 10.1016/0092-8674(90)90186-i

[bib10] Feeley KM, Fullard JF, Heneghan MA, Smith T, Maher M, Murphy RP, O'Gorman TA (1999) Microsatellite instability in sporadic colorectal carcinoma is not an indicator of prognosis. J Pathol 188: 14–17 10398134 10.1002/(SICI)1096-9896(199905)188:1<14::AID-PATH323>3.0.CO;2-Q

[bib11] Furlan D, Casati B, Cerutti R, Facco C, Terracciano L, Capella C, Chiaravalli AM (2002) Genetic progression in sporadic endometrial and gastrointestinal cancers with high microsatellite instability. J Pathol 197: 603–609 12210079 10.1002/path.1162

[bib12] Gafa R, Maestri I, Matteuzzi M, Santini A, Ferretti S, Cavazzini L, Lanza G (2000) Sporadic colorectal adenocarcinomas with high-frequency microsatellite instability. Cancer 89: 2025–2037 11066042

[bib13] Goel A, Arnold CN, Niedzwiecki D, Chang DK, Ricciardiello L, Carethers JM, Dowell JM, Wasserman L, Compton C, Mayer RJ, Bertagnolli MM, Boland CR (2003) Characterization of sporadic colon cancer by patterns of genomic instability. Cancer Res 63: 1608–1614 12670912

[bib14] Graham DM, Appelman HD (1990) Crohn's-like lymphoid reaction and colorectal carcinoma: a potential histologic prognosticator. Mod Pathol 3: 332–335 2362940

[bib15] Greenson JK, Bonner JD, Ben-Yzhak O, Cohen HI, Miselevich I, Resnick MB, Trougouboff P, Tomsho LD, Kim E, Low M, Almog R, Rennert G, Gruber SB (2003) Phenotype of microsatellite unstable colorectal carcinomas: well-differentiated and focally mucinous tumors and the absence of dirty necrosis correlate with microsatellite instability. Am J Surg Pathol 27: 563–570 12717242 10.1097/00000478-200305000-00001

[bib16] Gryfe R, Kim H, Hsieh ET, Aronson MD, Holowaty EJ, Bull SB, Redston M, Gallinger S (2000) Tumor microsatellite instability and clinical outcome in young patients with colorectal cancer. N Engl J Med 342: 69–77 10631274 10.1056/NEJM200001133420201

[bib17] Guidoboni M, Gafa R, Viel A, Doglioni C, Russo A, Santini A, Del Tin L, Macri E, Lanza G, Boiocchi M, Dolcetti R (2001) Microsatellite instability and high content of activated cytotoxic lymphocytes identify colon cancer patients with a favorable prognosis. Am J Pathol 159: 297–304 11438476 10.1016/S0002-9440(10)61695-1 PMC1850401

[bib18] Harrell FE (2001) Regression Modelling Strategies: With Applications to Linear Models, Logistic Regression, and Survival Analysis. Berlin: Springer

[bib19] Kane MF, Loda M, Gaida GM, Lipman J, Mishra R, Goldman H, Jessup JM, Kolodner R (1997) Methylation of the hMLH1 promoter correlates with lack of expression of hMLH1 in sporadic colon tumors and mismatch repair-defective human tumor cell lines. Cancer Res 57: 808–811 9041175

[bib20] Korn EL (1986) Censoring distributions as a measure of follow-up in survival analysis. Stat Med 5: 255–260 3738291 10.1002/sim.4780050306

[bib21] Lengauer C, Kinzler KW, Vogelstein B (1997) Genetic instability in colorectal cancers. Nature 386: 623–627 9121588 10.1038/386623a0

[bib22] Linnebacher M, Gebert J, Rudy W, Woerner S, Yuan YP, Bork P, von Knebel Doeberitz M (2001) Frameshift peptide-derived T-cell epitopes: a source of novel tumor-specific antigens. Int J Cancer 93: 6–11 11391614 10.1002/ijc.1298

[bib23] Lynch HT, de la Chapelle A (2003) Hereditary colorectal cancer. N Engl J Med 348: 919–932 12621137 10.1056/NEJMra012242

[bib24] Malkhosyan S, Rampino N, Yamamoto H, Perucho M (1996) Frameshift mutator mutations. Nature 382: 499–500 8700220 10.1038/382499a0

[bib25] Markowitz S, Wang J, Myeroff L, Parsons R, Sun LZ, Lutterbaugh J, Fan RS, Zborowska E, Kinzler KW, Vogelstein B, Brattaln M, Willson JK (1995) Inactivation of the type II TGF-beta receptor in colon cancer cells with microsatellite instability. Science 268: 1336–1338 7761852 10.1126/science.7761852

[bib26] Miyakura Y, Sugano K, Konishi F, Ichikawa A, Maekawa M, Shitoh K, Igarashi S, Kotake K, Koyama Y, Nagai H (2001) Extensive methylation of hMLH1 promoter region predominates in proximal colon cancer with microsatellite instability. Gastroenterology 121: 1300–1309 11729109 10.1053/gast.2001.29616

[bib27] O'Connell JB, Maggard MA, Liu JH, Etzioni DA, Livingston EH, Ko CY (2003) Rates of colon and rectal cancers are increasing in young adults. Am Surg 69: 866–872 14570365

[bib28] R Development Core Team (2003) R: A Language and Environment for Statistical Computing. Vienna, Austria: R Foundation for Statistical Computing

[bib29] Ribic CM, Sargent DJ, Moore MJ, Thibodeau SH, French AJ, Goldberg RM, Hamilton SR, Laurent-Puig P, Gryfe R, Shepherd LE, Tu D, Redston M, Gallinger S (2003) Tumor microsatellite instability status as a predictor of benefit from fluorouracil-based adjuvant chemotherapy for colon cancer. N Engl J Med 349: 247–257 12867608 10.1056/NEJMoa022289 PMC3584639

[bib30] Ripberger E, Linnebacher M, Schwitalle Y, Gebert J, von Knebel Doeberitz M (2003) Identification of HLA-A0201-restricted CTL epitope generated by a tumor-specific frameshift mutation in a coding microsatellite of the OGT gene. J Clin Immunol 23: 415–423 14601650 10.1023/a:1025329819121

[bib31] Rodriguez-Bigas MA, Boland CR, Hamilton SR, Henson DE, Jass JR, Khan PM, Lynch H, Perucho M, Smyrk T, Sobin L, Srivastava S (1997) A national cancer institute workshop on hereditary nonpolyposis colorectal cancer syndrom: meeting highlights and Bethesda guidelines. J Natl Cancer Inst 89: 1758–1762 9392616 10.1093/jnci/89.23.1758

[bib32] Ruschoff J, Roggendorf B, Brasch F, Mathiak M, Aust DE, Plaschke J, Mueller W, Poremba C, Kloor M, Keller G, Muders M, Blasenbreu-Vogt S, Rummele P, Muller A, Buttner R, Collaborative German Study Group on hereditary colorectal cancer funded by the German Cancer Aid (Deutsche Krebshilfe (2004) Molecular pathology in hereditary colorectal cancer. [Recommendations of the Collaborative German Study Group on hereditary colorectal cancer funded by the German Cancer Aid (Deutsche Krebshilfe)]. Pathologe 25: 178–192 15138699 10.1007/s00292-003-0641-x

[bib33] Saeterdal I, Gjertsen MK, Straten P, Eriksen JA, Gaudernack G (2001) A TGF betaRII frameshift-mutation-derived CTL epitope recognised by HLA-A2-restricted CD8+ T cells. Cancer Immunol Immunother 50: 469–476 11761441 10.1007/s002620100222 PMC11034255

[bib34] Salahshor S, Kressner U, Fischer H, Lindmark G, Glimelius B, Pahlman L, Lindblom A (1999) Microsatellite instability in sporadic colorectal cancer is not an independent prognostic factor. Br J Cancer 81: 190–193 10496341 10.1038/sj.bjc.6690676 PMC2362857

[bib35] Samowitz WS, Curtin K, Ma KN, Schaffer D, Coleman LW, Leppert M, Slattery ML (2001) Microsatellite instability in sporadic colon cancer is associated with an improved prognosis at the population level. Cancer Epidemiol Biomarkers Prev 10: 917–923 11535541

[bib36] Shia J, Ellis NA, Paty PB, Nash GM, Qin J, Offit K, Zhang XM, Markowitz AJ, Nafa K, Guillem JG, Wong WD, Gerald WL, Klimstra DS (2003) Value of histopathology in predicting microsatellite instability in hereditary nonpolyposis colorectal cancer and sporadic colorectal cancer. Am J Surg Pathol 27: 1407–1417 14576473 10.1097/00000478-200311000-00002

[bib37] Smyrk TC, Watson P, Kaul K, Lynch HT (2001) Tumor-infiltrating lymphocytes are a marker for microsatellite instability in colorectal carcinoma. Cancer 91: 2417–2422 11413533

[bib38] Sutter C, Gebert J, Bischoff P, Herfarth C, von Knebel Doeberitz M (1999) Molecular screening of potential HNPCC patients using a multiplex microsatellite PCR system. Mol Cell Probes 13: 157–165 10208807 10.1006/mcpr.1999.0231

[bib39] Turnbull Jr RB (1975) Current concepts in cancer. Cancer of the GI tract: colon, rectum, anus. The no-touch isolation technique of resection. JAMA 231: 1181–1182 1172824 10.1001/jama.231.11.1181

[bib40] Vasen HF, Mecklin JP, Meera Kahn P, Lynch HT (1991) The International Collaborative Group on Hereditary Non-Polyposis Colorectal Cancer (ICG-HNPCC). Dis Colon Rectum 34: 424–425 2022152 10.1007/BF02053699

[bib41] Watson P, Lin KM, Rodriguez-Bigas MA, Smyrk T, Lemon S, Shashidharan M, Franklin B, Karr B, Thorson A, Lynch HT (1998) Colorectal Carcinoma Survival among Hereditary Nonpolyposis Colorectal Carcinoma Family Members. Cancer 83: 259–266 9669808

[bib42] Woerner SM, Gebert J, Yuan YP, Sutter C, Ridder R, Bork P, von Knebel Doeberitz M (2003) Systematic identification of genes with coding microsatellites mutated in DNA mismatch repair-deficient cancer cells. Int J Cancer 93: 12–19 10.1002/ijc.1299 11391615

